# In This Issue

**DOI:** 10.1002/cfg.142

**Published:** 2002-02


**Comparative and Functional Genomics**
*Comp Funct Genom* 2002; **3**: 1–2.
**DOI:** 10.1002/cfg.142

### In this issue

### Expression profiling comparison of three *E.coli* DNA repair mutants with wild-type

Camilla Salmelin and Juhani Vilpo report on a study comparing three DNA repair-deficient substrains of *Escherichia coli* K12 with wild-type. The paper includes a detailed discussion of the statistical considerations for the analysis of microarray data. Although some significant differences were found, none of them have as yet been shown to relate to a conceivable consequence of the mutations, or compensation for the mutations.

### Special Section On The ESF 'Integrated Approaches For Functional Genomics' Programme Workshop 'Integrating Data In Genomics And Proteomics'

### Conference Report

Pierre-Alain Binz *et al.* and Antoine de Daruvar provide an in-depth report on the meeting, including some interesting insights from the discussion sessions.

### Conference Reviews

**Denis Shields** and **Aisling O'Halloran** review the considerations for integrating genotypic data with transcriptomic and proteomic data. These high-throughput approaches have the potential to identify the consequences of genetic variability. Studies combining these methods with genetic analyses could advance our understanding of the genetic aetiology of complex disorders

**Francisco Azuaje** gives us an overview of the problems currently being investigated by the computational intelligence community in working towards the semantic web, and discusses the potential for machine learning to aid the integration of data in the post-genomics era.

**Uwe Kärst *et al.*** describe the REALIS project, for post-genomic analysis of the food-born pathogen *Listeria monocytogenes*. The consortium is organised into work packages covering transcriptome and proteome profiling, analysis of regulatory elements, mutagenesis, comparative genomics and bioinformatics.

**Peter Rice *et al.*** present RibDB, an SRS-based database designed to meet the requirements of the REALIS consortium. RibDB integrates consortium data with that from public databases and provides a range of analysis applications to consortium members.

**Colin Harwood** and **Ivan Moszer** describe the BACELL project, for functional genomics of *Bacillus subtilis*. This project is generating data from a variety of functional genomics projects and gene regulation programmes. This data needs to be integrated with the vast array of biochemical and physiological data already available on this interesting bacterium. They discuss the challenges ahead and the work undertaken so far to meet them.

In his review on ontology-based document enrichment in bioinformatics, **Robert Stevens** discusses the uses of controlled vocabularies and how they can increase the utility of documents by making them computationally accessible.

**Henning Hermjakob** and **Rolf Apweiler** present Temblor, an EU funded project, which will provide an integrated view of genomic and proteomic data, drawn from databases across Europe. This will include the creation of new resources for protein interaction, structural and microarray data.

### Section on Pharmacogenetics

### Pyrosequencing for SNP detection

Mostafa Ronaghi and Elahe Elahi discuss the application of Pyrosequencing technology to mutation scanning, SNP discovery, and haplotyping.

### High-throughout SNP genotyping technologies

Suzanne Jenkins and Tim Gibson discuss the advantages and drawbacks of the chemistries and platforms available for high-throughput SNP genotyping. They also discuss potential routes to future increases in throughput. This is a critical requirement for whole genome approaches using SNPs, which could transform complex disease genetics and expedite pharmacogenetics research.

Copyright © 2002 John Wiley & Sons, Ltd.

### SNP and Mutation data on the Web

Mike Barnes reviews the resources currently available on the Internet for working with SNPs and mutations. There are two main databases acting as SNP repositories, dbSNP and HGBASE, which have different approaches, but do synchronise their datasets. There are also tools for SNP visualisation and mapping, including Ensembl and LocusLink. The mutation databases HGMD, OMIM and GDB are also profiled.

### Website Update: JEMBOSS

Tim Carver and Lisa Mullan present JEMBOSS, a Java interface for EMBOSS (The European Molecular Biology Open Software Suite). This suite of tools ranges from motif searches and sequence alignments to phylogenetic analyses and structural predictions.

Copyright © 2002 John Wiley & Sons, Ltd.

